# Receiver operating characteristic (ROC) curve for classification of ^18^F-NaF uptake on PET/CT*

**DOI:** 10.1590/0100-3984.2014.0119

**Published:** 2016

**Authors:** Agnes Araujo Valadares, Paulo Schiavom Duarte, Giovanna Carvalho, Carla Rachel Ono, George Barberio Coura-Filho, Heitor Naoki Sado, Marcelo Tatit Sapienza, Carlos Alberto Buchpiguel

**Affiliations:** 1 Nuclear Physician, Hospital das Clínicas da Faculdade de Medicina da Universidade de São Paulo (HC-FMUSP), São Paulo, SP, Brazil.; 2 PhD, Physician Assistant, Service of Nuclear Medicine - Instituto do Câncer do Estado de São Paulo Octavio Frias de Oliveira (Icesp), São Paulo, SP Brazil.; 3 Physician Assistant, Service of Nuclear Medicine - Instituto do Câncer do Estado de São Paulo Octavio Frias de Oliveira (Icesp), São Paulo, SP Brazil.; 4 Private Docent, Professor, Department of Radiology and Oncology - Faculdade de Medicina da Universidade de São Paulo (FMUSP), São Paulo, SP Brazil.; 5 Private Docent, Full Professor, Department of Radiology and Oncology - Faculdade de Medicina da Universidade de São Paulo (FMUSP), São Paulo, SP Brazil.

**Keywords:** ^18^F-NaF PET/CT, ROC curve, Cutoff values, Normal uptake, Malignant uptake

## Abstract

**Objective:**

To assess the cutoff values established by ROC curves to classify ^18^F-NaF uptake as normal or malignant.

**Materials and Methods:**

PET/CT images were acquired 1 hour after administration of 185 MBq of ^18^F-NaF. Volumes of interest (VOIs) were drawn on three regions of the skeleton as follows: proximal right humerus diaphysis (HD), proximal right femoral diaphysis (FD) and first vertebral body (VB1), in a total of 254 patients, totalling 762 VOIs. The uptake in the VOIs was classified as normal or malignant on the basis of the radiopharmaceutical distribution pattern and of the CT images. A total of 675 volumes were classified as normal and 52 were classified as malignant. Thirty-five VOIs classified as indeterminate or nonmalignant lesions were excluded from analysis. The standardized uptake value (SUV) measured on the VOIs were plotted on an ROC curve for each one of the three regions. The area under the ROC (AUC) as well as the best cutoff SUVs to classify the VOIs were calculated. The best cutoff values were established as the ones with higher result of the sum of sensitivity and specificity.

**Results:**

The AUCs were 0.933, 0.889 and 0.975 for UD, FD and VB1, respectively. The best SUV cutoffs were 9.0 (sensitivity: 73%; specificity: 99%), 8.4 (sensitivity: 79%; specificity: 94%) and 21.0 (sensitivity: 93%; specificity: 95%) for UD, FD and VB1, respectively.

**Conclusion:**

The best cutoff value varies according to bone region of analysis and it is not possible to establish one value for the whole body.

## INTRODUCTION

Sodium fluoride (^18^F-NaF) is a highly sensitive boneseeking PET tracer used to detect skeletal abnormalities. The uptake mechanism of ^18^F-fluoride resembles that of ^99m^Tc- MDP with better pharmacokinetic characteristics including faster blood clearance and two-fold higher uptake in bone^(1)^.

Over the last years there has been a renewed clinical interest in the use of ^18^F-NaF as a bone scanning agent^(2)^. Reasons for this resurgence include periodic worldwide shortages of ^99m^Tc needed for conventional bone scanning agents^(3)^, and the improved sensitivity^(4-6)^ and quantitative potential of ^18^F-NaF PET/CT^(7,8)^ as compared with ^99m^Tc-based conventional bone scans. In a pilot study, ^18^F-NaF was also demonstrated to be superior to ^18^F-FDG PET/CT and magnetic resonance imaging (MRI)^(9)^.

Standardized uptake value (SUV), which averages tracer uptake with respect to the injected dose and body weight, is the most widely used PET index for assessment of tracer uptake in the routine clinical practice because it does not require blood sampling and is obtained by static PET acquisition^(10-12)^. Although SUV has been used predominantly for ^18^F-FDG PET imaging quantification, research reports demonstrate that SUVs can detect significant metabolic change in individual metastatic lesions at ^18^F-NaF PET images, even in cases where visual evaluation reveals little if any difference^(7)^. Additionally, in the field of oncology, an earlier identification of metastatic involvement is feasible and SUV measurement may provide such information in cases where it is important to assess whether a patient is responding to treatment^(7,8)^.

However, as far as we know, the normal SUV range for ^18^F-NaF PET/CT has been rarely analyzed in the scientific literature^(13)^ as well as the most appropriate cutoff values to distinguish normal from malignant bone uptake.

The present study was aimed at establishing the normal SUV range for ^18^F-NaF PET/CT and assessing the most appropriate cutoff values to classify ^18^F-NaF uptake as normal or malignant.

## MATERIALS AND METHODS

### Patient population

The present study was approved by the committee for Ethics in Research of School of Medicine of Universidade de São Paulo.

In this cross-sectional study, the images of the first 254 patients submitted to ^18^F-NaF PET/CT scans in the authors' department were analyzed. The following patients' characteristics were analyzed: weight, height, body mass index (BMI), age and sex.

Main tumor types were the following: 96, breast cancer; 28, prostate cancer; 16, lung cancer; 9, colorectal cancer; 8, melanoma; 8, liver cancer. Thirty-four patients presented with other tumors and in 55 patients the cancer type was not recorded.

### PET/CT image acquisition

The patients were injected with 111 to 203 MBq (mean, 141 MBq) of ^18^F-NaF. Approximately 60 min after injection, all patients underwent whole-body (vertex to toes) three-dimensional PET/CT. Images were acquired on a Discovery 690 GE with the time of flight technique (General Electric). The PET image reconstruction was performed using iterative technique with 24 subsets for all studies. Low-dose CT transmission scans were obtained (10 to 30 mAs) for attenuation correction. Other CT images parameters were the following: 120 kVp, 0.5-s rotation time; 1.375 pitch; and axial slice thickness of 3.75 mm. Emission PET images were obtained at 1 min per bed position (15 cm slice thickness with 3 cm of overlapping), with 13 to 15 bed positions per study.

### Image analysis

**Figure 1:**
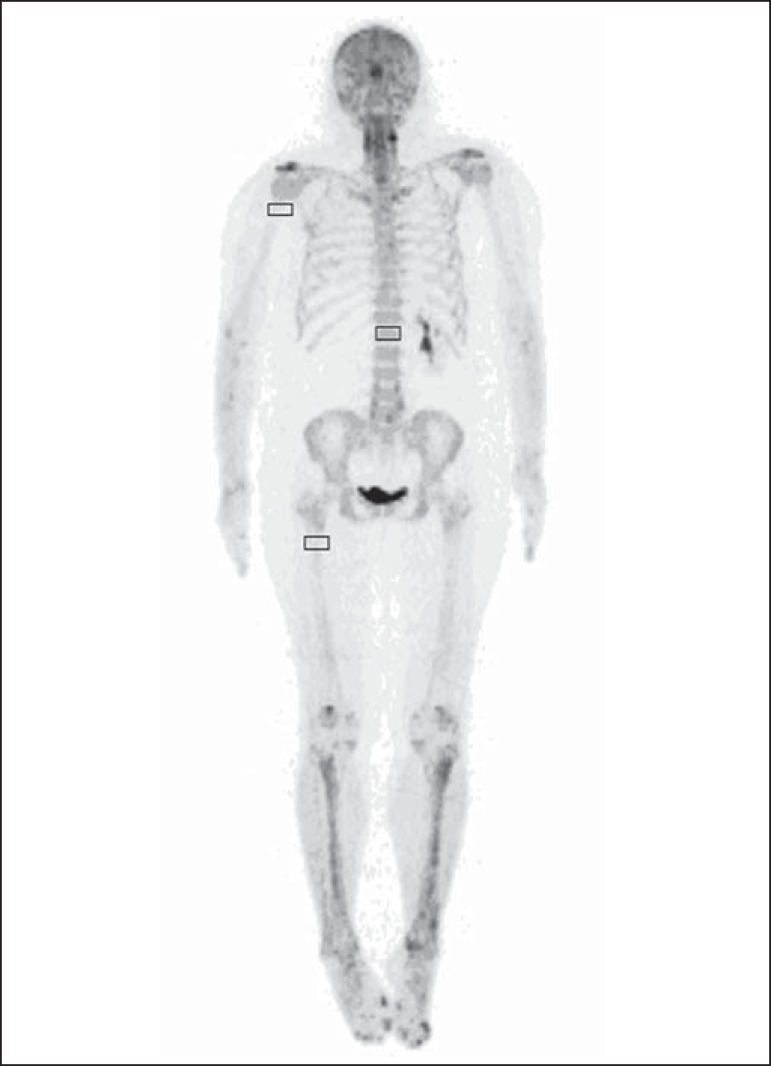
Example of VOIs drawn on three bone regions: proximal right humerus diaphysis; proximal right femoral diaphysis; and first vertebral body.

Cuboid volumes of interest (VOIs) with edges around 2 to 3 cm were drawn on three bone regions, as follows: proximal right humerus diaphysis (HD), proximal right femoral diaphysis (FD) and first vertebral body (VB1) in the 254 patients, totalling 762 VOIs (Figure 1). The uptake in the VOIs was classified as normal or malignant on the basis of the radiopharmaceutical distribution pattern and on the CT images. Such a classification was established by consensus between two nuclear medicine physicians. A total of 675 volumes were classified as normal, and 52 were classified as malignant. Thirty-five VOIs classified as indeterminate or nonmalignant lesions were excluded from analysis.

### Statistical analysis

For the whole study sample, the patients' characteristics (weight, height, BMI, age and sex) were described using mean and standard deviation.

The means and standard deviations of the maximal SUVs in each one of the three VOIs were calculated for the normal and malignant uptakes. ANOVA analysis was used to compare the maximal SUVs means among the three VOIs. The Tukey test was used to compare the maximal SUVs means between all pairs of regions.

The SUVs were also plotted on an ROC curve for each one of the three VOIs. The area under the ROC (AUC) as well as the most appropriate cutoff SUVs were calculated to classify the VOIs either as normal or malignant. The most appropriate cutoff values were established as the ones with higher result of the sum of sensitivity and specificity.

The statistical analyses were performed using Excel® worksheets and the SPSS 16.0®.

## RESULTS

The authors evaluated 254 patients, 66% of them, women. Mean and standard deviation of weight and height were, respectively, 69 ± 15 kg and 159 ± 8 cm. The group had a mean BMI of 27 ± 6 kg/m^2^ and an average age of 60 ± 14 years.

Means and the standard deviations of the maximum SUVs for each one of the three VOIs for normal and malignant uptakes are shown on Table 1.

**Table 1:** Mean ± standard deviation (SD) of maximum SUVs in the three bone regions – proximal right humerus diaphysis (HD), proximal right femoral diaphysis (FD) and first vertebral body (VB1) – for normal and malignant classification

VOI	Classification	*n*	SUV (mean ± SD)
VB1	Normal	207	14.4 ± 3.7
VB1	Malignant	27	39.3 ± 18.6
HD	Normal	236	3.8 ± 1.4
HD	Malignant	11	11.4 ± 4.9
FD	Normal	232	5.4 ± 2.0
FD	Malignant	14	14.4 ± 9.0

The ANOVA analysis comparing the means among the three VOIs in the regions classified as normal demonstrated a statistically significant difference (*p* < 0.01). The Tukey test demonstrated a statistically significant difference between all pairs of regions as compared with each other (*p* < 0.01).

**Figure 2:**
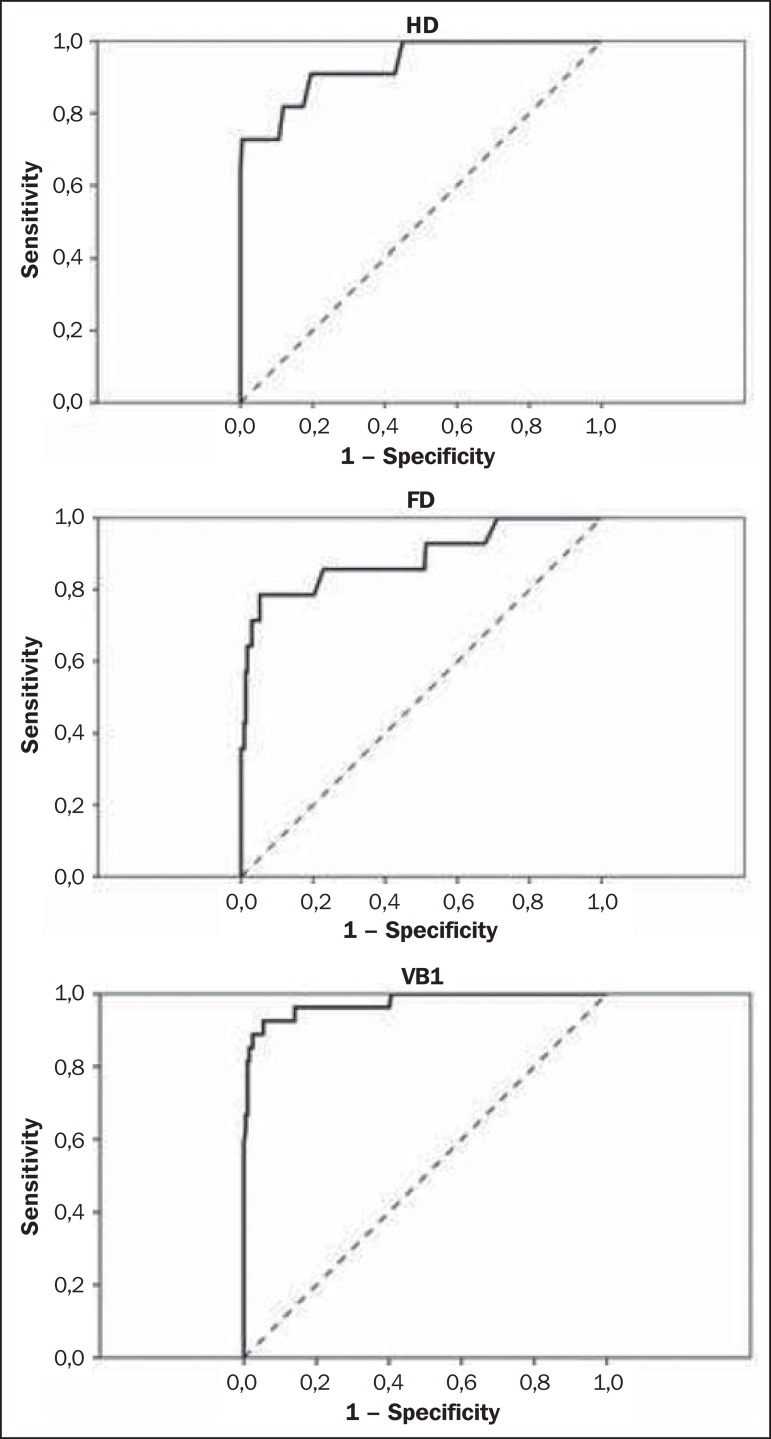
The ROC curves for the three bone regions: proximal right humerus diaphysis (HD), proximal right femoral diaphysis (FD) and first vertebral body (VB1).

The AUCs were 0.933, 0.889 and 0.975 for UD, FD and VB1, respectively (Figure 2). The most appropriate SUV cutoffs were 9.0 (sensitivity: 73%; specificity: 99%), 8.4 (sensitivity: 79%; specificity: 94%), and 21 (sensitivity: 93%; specificity: 95%) for UD, FD and VB1, respectively.

## DISCUSSION

^18^F-NaF was introduced by Blau et al.^(14)^ in 1962 as an imaging agent for bone lesions. Data from multiple small studies have shown that ^18^F-NaF PET produces bone scans with higher sensitivity and specificity than ^99m^Tc-based bone scans^(15-19)^, and has also demonstrated to be superior to ^18^FFDG PET/CT and MRI^(9)^.

The SUV is commonly used as a relative measure of FDG uptake^(20)^. The use of SUV is currently a routine in the oncological clinical practice of ^18^FFDG PET/CT imaging. Ideally, the use of SUV removes the variability caused by differences in patients' size and the amount of injected activity^(21)^ and facilitates comparisons between patients.

Although the practice of using SUV thresholds for diagnosis is not widely accepted^(22)^ and despite the fact that it has been repeatedly demonstrated that the use of SUV thresholds characterizes an uptake region as benign or malignant is often invalid, there are some situations where the use of SUV may be useful^(21)^. The SUV could be useful in cases where PET quantitative imaging is necessary in clinical research, clinical trials, and researches for development of drugs since it allows the standardization of imaging analysis^(23)^. The SUV could also be useful in therapy monitoring scans as independent measures of changes in metabolic activity can provide an alternative approach to assess response to therapy^(21)^. Even for diagnostic purposes, the use of SUV thresholds could be valuable in some situations. For example, in cases where the FDG uptake in a tissue is no greater than the uptake in adjacent reference tissue and where the pre-test likelihood of malignancy is low, it is considered to be safe to adopt an expectant strategy^(24)^.

Most of the studies approaching the use of SUV in nuclear medicine were developed for ^18^FFDG PET/CT, even in the field of skeletal pathologies^(25)^. However, recent reports describe the use of SUV to analyze bone pathologies at ^18^FNaF PET/CT. A recent scientific article has demonstrated that ^18^F-fluoride PET scans using SUV measurements have the potential to be a diagnostic tool in otosclerosis^(26)^. It was also demonstrated that SUV can be considered as being as effective and accurate as kinetic modeling in measuring the response of pagetic bones to bisphosphonates by means of ^18^F-fluoride PET^(27)^. Despite these studies, there is still little experience with the use of reference values of SUV in bone metabolism^(13)^.

In the present study, the authors analyzed the normal range of SUVs in a group of patients as well as tried to define the most appropriate values to distinguish between normal and malignant uptakes. The authors have observed that the normal SUVs range varies among the analyzed bone regions and that it is lower in the humerus than in the femur, additionally, it is lower in these two bones as compared with the first lumbar vertebral body. The authors also observed that, although the AUC is high in the three regions, the most appropriate cutoff value to classify bone uptake as normal or malignant varies among these regions and it is not possible to establish one single value for the whole body.

In a previous study, Win et al. have analyzed the ^18^F-fluoride maximum SUVs in the skeleton^(13)^. Their findings were similar to the findings of the present study. According to their study, various skeletal sites have different normal SUVs and vertebral bodies tend to show the highest values. They deeply discussed the causes of these differences in the SUVs and related some possible explanations as a higher flow in the spine as compared with the proximal femur^(28)^, a greater bone turnover at the spine than at other skeletal sites^(29)^, predominance of cortical bone in the humerus that has a lower level of bone metabolism as compared with the lumbar spine which is rich in trabecular bone^(30)^, and the mechanical stress that lumbar vertebra is subjected as it is a primary weight bearing bone^(31)^. However, their mean SUVs in the same bone regions analyzed in our study are lower: 7.16 in the VB1, 2.16 in the femur and 1,71 in the humerus. The reasons for such differences between the two studies are still to be known and should be an object of further analysis. But, one must note that SUVs also depend on the measurement instruments and reconstruction methods^(32,33)^; therefore, differences between the images acquisition and reconstruction protocols could partially explain such discrepancies. A more recent study developed by Sabbah et al.^34^ also analyzed ^18^F-fluoride SUVs in the skeleton. They found mean values of 10.07, 2.12 and 3.01 for maximum SUVs in the lumbar spine, humerus and femur, respectively. These values are also compatible with the results of the present study as they demonstrated that the mean SUVs vary among different bone structures and that the SUV in the lumbar spine is higher than the ones in the femur and humerus. Once again, the absolute values obtained are different from our results but they are also different from the values obtained by Win et al.^(13)^. The reasons for such differences between the three studies should be the same discussed above: variability in the images acquisition and reconstruction protocols.

Finally, an important aspect to be discussed is the use of ROC curves and threshold values in the present study. ROC curves were used in two aspects, namely, to define the most appropriate threshold to differentiate malignant from normal uptake areas in the three bone regions and to analyze the overall ^18^F-fluoride PET/CT accuracy to differentiate malignant from normal uptake in those regions. The definition of the thresholds was important to demonstrate that those values vary among the analyzed regions and corroborated the authors' findings that the normal SUVs range should be established for each one of the bone regions. However, is important to observe that the threshold values established in the present study should not be used to classify lesions as malignant since we did not analyze the SUVs in a group of benign lesions. Moreover, in the authors' clinical experience, the ^18^F-fluoride uptake intensity in benign bone lesions could be as intense as the uptake in malignant lesions, and some malignant lesions may present a very low uptake. Therefore, for the time being, SUVs in ^18^F-fluoride PET/CT studies should be used at most for follow-up purposes and not to classify the lesions as malign or benign.

## CONCLUSION

The SUVs normal range and the most appropriate cutoff value to differentiate normal from malignant bone uptake vary according to bone region of analysis and it is not possible to establish a single value for the whole skeleton.
